# *trans*-5,6-Diphenyl­perhydro­pyran-2,4-dione

**DOI:** 10.1107/S1600536809000087

**Published:** 2009-01-08

**Authors:** Laura C. de Souza, Dennis de O. Imbroisi, Carlos A. De Simone, Mariano A. Pereira, Valéria R. S. Malta

**Affiliations:** a Instituto de Química e Biotecnologia, Universidade Federal de Alagoas, 57072-970 Maceió, AL, Brazil

## Abstract

In the title compound, C_17_H_14_O_3_, the pyran ring adopts a boat conformation and the dihedral angle between the aromatic ring planes is 59.1 (1)°. In the crystal structure inter­molecular C—H⋯O hydrogen bonds and C—H⋯π inter­actions link the mol­ecules.

## Related literature

For general background, see: Yen & Chen (1995 ▶); Soler-Rivas *et al.* (2000 ▶). For related structures and biological activity, see: Brand-William *et al.* (1995 ▶); Sánchez-Moreno *et al.* (1998 ▶); Souza *et al.* (2004 ▶). For the synthesis, see: Souza (2008 ▶). For geometric analysis, see: Cremer & Pople (1975 ▶). For bond-length data, see: Allen *et al.* (1987 ▶).
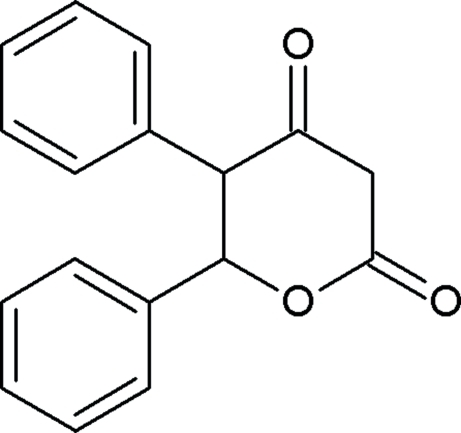


## Experimental

### 

#### Crystal data

C_17_H_14_O_3_
*M* *_r_* = 266.28
Monoclinic, 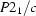

*a* = 8.9940 (2) Å
*b* = 8.2310 (4) Å
*c* = 18.9040 (8) Å
β = 101.412 (2)°
*V* = 1371.79 (9) Å^3^
*Z* = 4
Mo *K*α radiation
μ = 0.09 mm^−1^
*T* = 295 K
0.30 × 0.30 × 0.18 mm

#### Data collection

Nonius KappaCCD diffractometer
Absorption correction: none
5298 measured reflections
3113 independent reflections
2459 reflections with *I* > 2σ(*I*)
*R*_int_ = 0.017

#### Refinement

*R*[*F* ^2^ > 2σ(*F* ^2^)] = 0.050
*wR*(*F* ^2^) = 0.131
*S* = 1.05
3113 reflections
181 parameters
H-atom parameters constrained
Δρ_max_ = 0.19 e Å^−3^
Δρ_min_ = −0.20 e Å^−3^

### 

Data collection: *COLLECT* (Nonius, 2000 ▶); cell refinement: *SCALEPACK* (Otwinowski & Minor, 1997 ▶); data reduction: *DENZO* (Otwinowski & Minor, 1997 ▶) and *SCALEPACK*; program(s) used to solve structure: *SHELXS97* (Sheldrick, 2008 ▶); program(s) used to refine structure: *SHELXL97* (Sheldrick, 2008 ▶); molecular graphics: *ORTEP-3 for Windows* (Farrugia, 1997 ▶); software used to prepare material for publication: *WinGX* (Farrugia, 1999 ▶).

## Supplementary Material

Crystal structure: contains datablocks I, global. DOI: 10.1107/S1600536809000087/bq2110sup1.cif

Structure factors: contains datablocks I. DOI: 10.1107/S1600536809000087/bq2110Isup2.hkl

Additional supplementary materials:  crystallographic information; 3D view; checkCIF report

## Figures and Tables

**Table 1:** Hydrogen-bond geometry (Å, °)

*D*—H⋯*A*	*D*—H	H⋯*A*	*D*⋯*A*	*D*—H⋯*A*
C6—H6⋯O2^i^	0.98	2.44	3.380 (2)	161
C17—H17⋯O3^ii^	0.93	2.46	3.351 (3)	160
C3—H3*B*⋯*Cg*1^i^	0.97	2.97	3.681 (2)	131
C5—H5⋯*Cg*2^iii^	0.98	2.96	3.830 (2)	149

Symmetry codes: (i) 
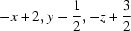
; (ii) 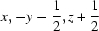; (iii) 
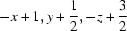
. *Cg*1 and *Cg*2 are the centroids of the C7–C12 and C13–C18 rings, respectively.

**Fig. 1.**
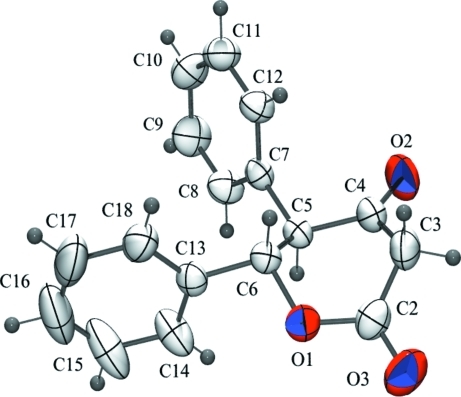
Projection of C17H14O3, showing the atom labelling with 50% probability displacement.

**Fig. 2.**
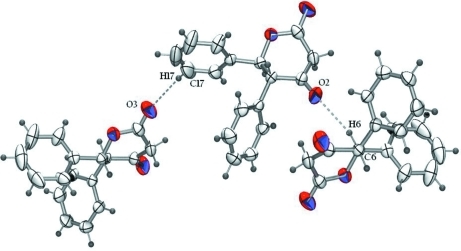
Hydrogen interactions.

## supplementary crystallographic information

### Comment

The free radicals generated in bioorganic redoxi processes induce oxidative damage in various components of the cells (*e.g.*, lipids, proteins and nucleic acids) and their play a significant role in the development of life-limiting chronic diseases such as cancer, hypertension, arteriosclerosis, rheumatism, cataracts and other (Yen & Chen, 1995; Soler-Rivas *et al.*,2000). The dihydropyran-2,4-diones exhibit structural features present bin many biologically active natural products possessing important pharmacological activities (Brand-William *et al.*,1995; Sánchez-Moreno *et al.*,1998). As part of our continuing studies aimed at ascertaining the biological activity of this class, the title compound was synthetized (Souza, 2008) and its antioxidant activity analyzed *in vitro*, by measuring the decrease in absorbance at 515 nm that occurred when the 2,2-diphenyl-1-picryl-hydrazyl radical (DPPH) was reduced by the antioxidant. The spectrophotometric assay was used to determine the radical scavenging activity (Souza *et al.*,2004).

The *ORTEP-3* (Farrugia, 1997) representation of the title compound (5,6-DPDP) is showing in (Fig. 1). Bond lengths and angles are in good agreement with the expected values reported in the literature (Allen *et al.*, 1987). The pirane ring adopts a boat conformation and the calculated puckering parameters are: q_2_ = 0.624 (1) Å, q_3_ = 0.121 (1) Å, Q_T_ = 0.636 (1) Å, θ = 79.0 (1)° and φ = 287.5 (1)° (Cremer & Pople, 1975). The dihedral angle between planes passing through atoms C7—C12 and C13—C18 of the aromatic rings is 59.1 (1) °. In the crystal packing, molecules interact through two intermolecular C–H···O hydrogen bonds and two C—H···.π interactions, Fig. 2 and Table 1.

### Experimental

The *trans*-5, 6-diphenyltetradehydropyran-2,4-dione has showed similar antioxidant activity at the positive control, the synthetic antioxidant BHT (2,6-di-*tert*-butyl-4-methylphenol) used as food conserving. The reduction percentage after 60 minutes to a solution of 20 nM of sample were 88% to 5,6-DPDP and 82% to BHT (Souza, 2008).The 5,6-DPDP was synthesized in one pot by preparation of the dianion of the ethyl 3-oxo-4-phenylbutanoate (NaH, n-butillithium, THF, -10° C), and alkylation reaction with benzaldehyde followed by ester hydrolysis (NaOH, H_2_O, 12 h, RT) and lactonization in acidic medium (HCl, H_2_O, 2 h, 0°C). The compound was purified by silica gel chromatography and the crystals for *x*-ray diffraction studies were grown by slow evaporation from a CHCl_3_ solution.

### Refinement

H atoms were located on stereochemical grounds and refined with fixed geometry, each riding on a carrier atom, with C—H = [0.93 - 0.98] Å and anisotropic displacement parameter amounting to 1.5 (for Methyl-H atoms) and 1.2 (for the other H atoms) times the value of the equivalent isotropic displacement parameter of the which they are attached.The maximum and minimum residual electron density peaks were located 0.73 and 0.74 Å, from the C5 and H15 atoms respectively.

### Crystal data

**Table:**

C_17_H_14_O_3_	*F*(000) = 560
*M**_r_* = 266.28	*D*_x_ = 1.289 Mg m^−^^3^
Monoclinic, *P*2_1_/*c*	Mo *K*α radiation, λ = 0.71073 Å
Hall symbol: -P2ybc	Cell parameters from 2880 reflections
*a* = 8.9940 (2) Å	θ = 1.0–27.5°
*b* = 8.2310 (4) Å	µ = 0.09 mm^−^^1^
*c* = 18.9040 (8) Å	*T* = 295 K
β = 101.412 (2)°	Prism, yellow
*V* = 1371.79 (9) Å^3^	0.30 × 0.30 × 0.18 mm
*Z* = 4	

### Data collection

**Table:**

Nonius KappaCCD diffractometer	2459 reflections with *I* > 2σ(*I*)
Radiation source: Enraf Nonius FR590	*R*_int_ = 0.017
horizonally mounted graphite crystal	θ_max_ = 27.5°, θ_min_ = 2.3°
Detector resolution: 9 pixels mm^-1^	*h* = −11→11
CCD rotation images, thick slices scans	*k* = −9→10
5298 measured reflections	*l* = −24→24
3113 independent reflections	

### Refinement

**Table:**

Refinement on *F*^2^	Primary atom site location: structure-invariant direct methods
Least-squares matrix: full	Secondary atom site location: difference Fourier map
*R*[*F*^2^ > 2σ(*F*^2^)] = 0.050	Hydrogen site location: inferred from neighbouring sites
*wR*(*F*^2^) = 0.131	H-atom parameters constrained
*S* = 1.05	*w* = 1/[σ^2^(*F*_o_^2^) + (0.0508*P*)^2^ + 0.3831*P*] where *P* = (*F*_o_^2^ + 2*F*_c_^2^)/3
3113 reflections	(Δ/σ)_max_ < 0.001
181 parameters	Δρ_max_ = 0.19 e Å^−^^3^
0 restraints	Δρ_min_ = −0.20 e Å^−^^3^

### Special details

**Table:**

Geometry. All e.s.d.'s (except the e.s.d. in the dihedral angle between two l.s. planes) are estimated using the full covariance matrix. The cell e.s.d.'s are taken into account individually in the estimation of e.s.d.'s in distances, angles and torsion angles; correlations between e.s.d.'s in cell parameters are only used when they are defined by crystal symmetry. An approximate (isotropic) treatment of cell e.s.d.'s is used for estimating e.s.d.'s involving l.s. planes.
Refinement. Refinement of *F*^2^ against ALL reflections. The weighted *R*-factor *wR* and goodness of fit *S* are based on *F*^2^, conventional *R*-factors *R* are based on *F*, with *F* set to zero for negative *F*^2^. The threshold expression of *F*^2^ > σ(*F*^2^) is used only for calculating *R*-factors(gt) *etc*. and is not relevant to the choice of reflections for refinement. *R*-factors based on *F*^2^ are statistically about twice as large as those based on *F*, and *R*- factors based on ALL data will be even larger.

### Fractional atomic coordinates and isotropic or equivalent isotropic displacement parameters (Å^2^)

**Table:**

	*x*	*y*	*z*	*U*_iso_*/*U*_eq_	
C2	0.69397 (19)	−0.0595 (2)	0.63794 (8)	0.0521 (4)	
C3	0.84834 (19)	0.0134 (2)	0.65634 (8)	0.0560 (4)	
H3A	0.8717	0.0620	0.6131	0.067*	
H3B	0.9211	−0.0730	0.6712	0.067*	
C4	0.86937 (16)	0.1403 (2)	0.71484 (8)	0.0476 (4)	
C5	0.76480 (14)	0.12492 (17)	0.76852 (7)	0.0383 (3)	
H5	0.6771	0.1953	0.7515	0.046*	
C6	0.70604 (15)	−0.05049 (17)	0.76642 (7)	0.0385 (3)	
H6	0.7925	−0.1246	0.7790	0.046*	
C7	0.83633 (14)	0.17992 (16)	0.84390 (7)	0.0385 (3)	
C8	0.76586 (17)	0.2946 (2)	0.87924 (9)	0.0520 (4)	
H8	0.6745	0.3402	0.8562	0.062*	
C9	0.8303 (2)	0.3421 (2)	0.94867 (10)	0.0652 (5)	
H9	0.7817	0.4189	0.9722	0.078*	
C10	0.9657 (2)	0.2763 (2)	0.98306 (9)	0.0631 (5)	
H10	1.0094	0.3097	1.0295	0.076*	
C11	1.03660 (18)	0.1616 (2)	0.94891 (9)	0.0565 (4)	
H11	1.1281	0.1166	0.9723	0.068*	
C12	0.97190 (16)	0.11267 (19)	0.87959 (8)	0.0477 (4)	
H12	1.0198	0.0340	0.8567	0.057*	
C13	0.59831 (15)	−0.07985 (17)	0.81644 (7)	0.0412 (3)	
C14	0.46566 (19)	0.0086 (2)	0.80971 (12)	0.0644 (5)	
H14	0.4401	0.0834	0.7724	0.077*	
C15	0.3711 (3)	−0.0137 (3)	0.85803 (16)	0.0935 (8)	
H15	0.2827	0.0473	0.8535	0.112*	
C16	0.4061 (3)	−0.1242 (4)	0.91217 (15)	0.1019 (10)	
H16	0.3420	−0.1378	0.9447	0.122*	
C17	0.5352 (3)	−0.2153 (4)	0.91893 (11)	0.0967 (9)	
H17	0.5583	−0.2915	0.9558	0.116*	
C18	0.6323 (2)	−0.1938 (2)	0.87041 (9)	0.0643 (5)	
H18	0.7197	−0.2564	0.8746	0.077*	
O1	0.62369 (12)	−0.08786 (13)	0.69346 (5)	0.0487 (3)	
O2	0.96257 (15)	0.24604 (19)	0.71798 (7)	0.0767 (4)	
O3	0.63076 (17)	−0.0935 (2)	0.57795 (6)	0.0792 (4)	

### Atomic displacement parameters (Å^2^)

**Table:**

	*U*^11^	*U*^22^	*U*^33^	*U*^12^	*U*^13^	*U*^23^
C2	0.0651 (10)	0.0484 (9)	0.0431 (8)	0.0015 (7)	0.0113 (7)	−0.0051 (7)
C3	0.0617 (9)	0.0655 (10)	0.0462 (8)	−0.0027 (8)	0.0239 (7)	−0.0015 (7)
C4	0.0423 (7)	0.0550 (9)	0.0467 (8)	−0.0069 (7)	0.0113 (6)	0.0061 (7)
C5	0.0347 (6)	0.0387 (7)	0.0422 (7)	−0.0028 (5)	0.0091 (5)	0.0005 (6)
C6	0.0368 (6)	0.0390 (7)	0.0393 (7)	−0.0005 (5)	0.0065 (5)	−0.0010 (5)
C7	0.0355 (6)	0.0375 (7)	0.0436 (7)	−0.0054 (5)	0.0105 (5)	−0.0013 (6)
C8	0.0456 (8)	0.0520 (9)	0.0578 (9)	0.0054 (7)	0.0089 (7)	−0.0089 (7)
C9	0.0683 (11)	0.0677 (11)	0.0606 (10)	0.0065 (9)	0.0158 (8)	−0.0212 (9)
C10	0.0674 (11)	0.0741 (12)	0.0456 (9)	−0.0053 (9)	0.0054 (7)	−0.0149 (8)
C11	0.0462 (8)	0.0640 (10)	0.0558 (9)	0.0005 (7)	0.0015 (7)	−0.0007 (8)
C12	0.0400 (7)	0.0506 (8)	0.0524 (8)	0.0012 (6)	0.0091 (6)	−0.0066 (7)
C13	0.0426 (7)	0.0381 (7)	0.0435 (7)	−0.0100 (5)	0.0100 (6)	−0.0046 (6)
C14	0.0544 (9)	0.0477 (9)	0.0998 (14)	−0.0010 (7)	0.0365 (9)	0.0020 (9)
C15	0.0823 (14)	0.0728 (14)	0.147 (2)	−0.0182 (11)	0.0759 (15)	−0.0221 (15)
C16	0.113 (2)	0.118 (2)	0.0933 (17)	−0.0592 (18)	0.0672 (16)	−0.0339 (16)
C17	0.1149 (19)	0.122 (2)	0.0517 (11)	−0.0542 (18)	0.0137 (12)	0.0191 (12)
C18	0.0620 (10)	0.0742 (12)	0.0529 (9)	−0.0146 (9)	0.0023 (8)	0.0170 (9)
O1	0.0522 (6)	0.0502 (6)	0.0433 (6)	−0.0104 (5)	0.0084 (4)	−0.0078 (5)
O2	0.0722 (8)	0.0922 (10)	0.0712 (8)	−0.0402 (7)	0.0275 (6)	−0.0053 (7)
O3	0.0974 (10)	0.0914 (10)	0.0454 (7)	−0.0122 (8)	0.0061 (6)	−0.0174 (7)

### Geometric parameters (Å, °)

**Table:**

C2—O3	1.1971 (19)	C9—C10	1.374 (3)
C2—O1	1.3484 (19)	C9—H9	0.9300
C2—C3	1.489 (2)	C10—C11	1.370 (2)
C3—C4	1.506 (2)	C10—H10	0.9300
C3—H3A	0.9700	C11—C12	1.384 (2)
C3—H3B	0.9700	C11—H11	0.9300
C4—O2	1.2013 (19)	C12—H12	0.9300
C4—C5	1.5191 (19)	C13—C18	1.375 (2)
C5—C7	1.5122 (18)	C13—C14	1.382 (2)
C5—C6	1.5353 (19)	C14—C15	1.378 (3)
C5—H5	0.9800	C14—H14	0.9300
C6—O1	1.4633 (16)	C15—C16	1.358 (4)
C6—C13	1.5013 (18)	C15—H15	0.9300
C6—H6	0.9800	C16—C17	1.367 (4)
C7—C8	1.381 (2)	C16—H16	0.9300
C7—C12	1.387 (2)	C17—C18	1.398 (3)
C8—C9	1.382 (2)	C17—H17	0.9300
C8—H8	0.9300	C18—H18	0.9300
			
O3—C2—O1	119.27 (16)	C10—C9—H9	119.9
O3—C2—C3	124.18 (16)	C8—C9—H9	119.9
O1—C2—C3	116.55 (13)	C11—C10—C9	120.03 (16)
C2—C3—C4	115.28 (13)	C11—C10—H10	120.0
C2—C3—H3A	108.5	C9—C10—H10	120.0
C4—C3—H3A	108.5	C10—C11—C12	119.91 (15)
C2—C3—H3B	108.5	C10—C11—H11	120.0
C4—C3—H3B	108.5	C12—C11—H11	120.0
H3A—C3—H3B	107.5	C11—C12—C7	120.60 (14)
O2—C4—C3	121.58 (14)	C11—C12—H12	119.7
O2—C4—C5	123.07 (15)	C7—C12—H12	119.7
C3—C4—C5	115.35 (12)	C18—C13—C14	119.27 (15)
C7—C5—C4	113.59 (11)	C18—C13—C6	120.03 (14)
C7—C5—C6	112.70 (11)	C14—C13—C6	120.69 (14)
C4—C5—C6	108.47 (11)	C15—C14—C13	120.3 (2)
C7—C5—H5	107.2	C15—C14—H14	119.9
C4—C5—H5	107.2	C13—C14—H14	119.9
C6—C5—H5	107.2	C16—C15—C14	120.5 (2)
O1—C6—C13	106.88 (10)	C16—C15—H15	119.8
O1—C6—C5	109.24 (11)	C14—C15—H15	119.8
C13—C6—C5	113.39 (11)	C15—C16—C17	120.2 (2)
O1—C6—H6	109.1	C15—C16—H16	119.9
C13—C6—H6	109.1	C17—C16—H16	119.9
C5—C6—H6	109.1	C16—C17—C18	119.9 (2)
C8—C7—C12	118.76 (13)	C16—C17—H17	120.0
C8—C7—C5	120.68 (13)	C18—C17—H17	120.0
C12—C7—C5	120.54 (13)	C13—C18—C17	119.8 (2)
C7—C8—C9	120.43 (15)	C13—C18—H18	120.1
C7—C8—H8	119.8	C17—C18—H18	120.1
C9—C8—H8	119.8	C2—O1—C6	118.00 (11)
C10—C9—C8	120.25 (16)		
			
O3—C2—C3—C4	141.51 (18)	C9—C10—C11—C12	0.3 (3)
O1—C2—C3—C4	−38.6 (2)	C10—C11—C12—C7	0.6 (3)
C2—C3—C4—O2	−153.41 (17)	C8—C7—C12—C11	−1.0 (2)
C2—C3—C4—C5	26.3 (2)	C5—C7—C12—C11	−179.25 (14)
O2—C4—C5—C7	−33.0 (2)	O1—C6—C13—C18	119.45 (14)
C3—C4—C5—C7	147.27 (13)	C5—C6—C13—C18	−120.13 (15)
O2—C4—C5—C6	−159.12 (16)	O1—C6—C13—C14	−61.63 (17)
C3—C4—C5—C6	21.12 (17)	C5—C6—C13—C14	58.80 (18)
C7—C5—C6—O1	173.61 (10)	C18—C13—C14—C15	2.1 (3)
C4—C5—C6—O1	−59.72 (13)	C6—C13—C14—C15	−176.85 (17)
C7—C5—C6—C13	54.54 (15)	C13—C14—C15—C16	−0.9 (3)
C4—C5—C6—C13	−178.80 (11)	C14—C15—C16—C17	−0.5 (4)
C4—C5—C7—C8	126.23 (15)	C15—C16—C17—C18	0.6 (4)
C6—C5—C7—C8	−109.89 (15)	C14—C13—C18—C17	−1.9 (3)
C4—C5—C7—C12	−55.59 (18)	C6—C13—C18—C17	176.99 (16)
C6—C5—C7—C12	68.29 (16)	C16—C17—C18—C13	0.6 (3)
C12—C7—C8—C9	0.5 (2)	O3—C2—O1—C6	177.79 (15)
C5—C7—C8—C9	178.72 (15)	C3—C2—O1—C6	−2.1 (2)
C7—C8—C9—C10	0.4 (3)	C13—C6—O1—C2	175.37 (12)
C8—C9—C10—C11	−0.9 (3)	C5—C6—O1—C2	52.33 (16)

### Hydrogen-bond geometry (Å, °)

**Table:**

*D*—H···*A*	*D*—H	H···*A*	*D*···*A*	*D*—H···*A*
C6—H6···O2^i^	0.98	2.44	3.380 (2)	161
C17—H17···O3^ii^	0.93	2.46	3.351 (3)	160
C3—H3B···Cg1^i^	0.97	2.98	3.681 (2)	131
C5—H5···Cg2^iii^	0.98	2.96	3.830 (2)	149

Symmetry codes: (i) −*x*+2, *y*−1/2, −*z*+3/2; (ii) *x*, −*y*−1/2, *z*+1/2; (iii) −*x*+1, *y*+1/2, −*z*+3/2.
